# Acral Prurigo Nodularis: A Case Report

**DOI:** 10.7759/cureus.29405

**Published:** 2022-09-21

**Authors:** Abdulaziz Albinhamad, Mohammad Almazied, Ru'aa Alharithy, Ahmed Alhumidi

**Affiliations:** 1 College of Medicine, Imam Mohammad Ibn Saud Islamic University, Riyadh, SAU; 2 Dermatology, Security Forces Hospital, Princess Nourah Bint Abdulrahman University, Riyadh, SAU; 3 Pathology, College of Medicine, King Saud University, Riyadh, SAU

**Keywords:** prurigo nodularis, itch, chronic pruritus, skin nodules, prurigo nodularis (pn), acral

## Abstract

Prurigo nodularis (PN) is a chronic skin disease that manifests with severe itchy, firm, hyperkeratotic nodules distributed on the trunk and the extremities symmetrically. Here, we report a unique presentation of PN. A 26-year-old male presented with multiple itchy nodules over the hands and feet sparing the trunk, which were confirmed histologically as PN. This is the first reported case of PN with exclusive acral distribution.

## Introduction

Prurigo nodularis (PN) is a skin disease that manifests with severe itchy lesions described as well-defined, scaly, firm, hyperkeratotic itchy nodules [1]. The lesions are usually distributed on the trunk and the extremities symmetrically. This disease is challenging to treat, resulting in psychological and physical impacts, which can affect the quality of life [2]. The diagnosis is usually clinical and supported by biopsy [3]. Herein, we present a case of PN with an unusual presentation, which is limited to hands and feet.

## Case presentation

**Figure 1:**
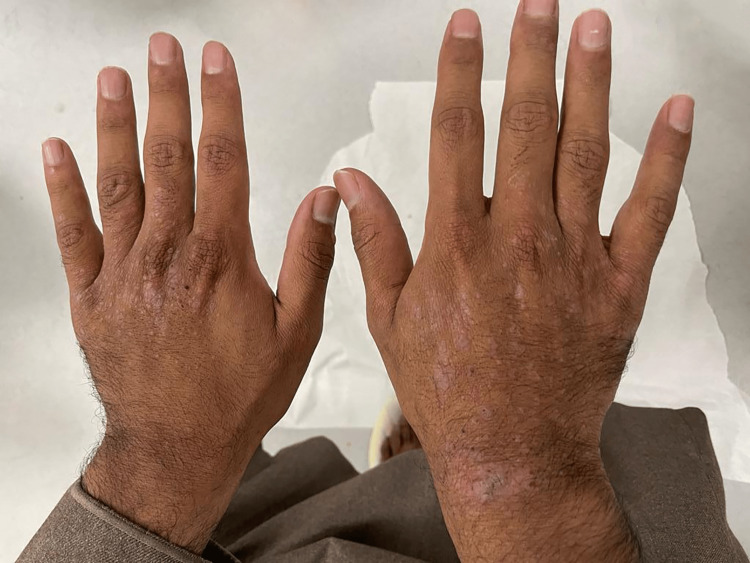
Multiple scaly, hyperkeratotic nodules and excoriations over the dorsal surface of both hands

**Figure 2:**
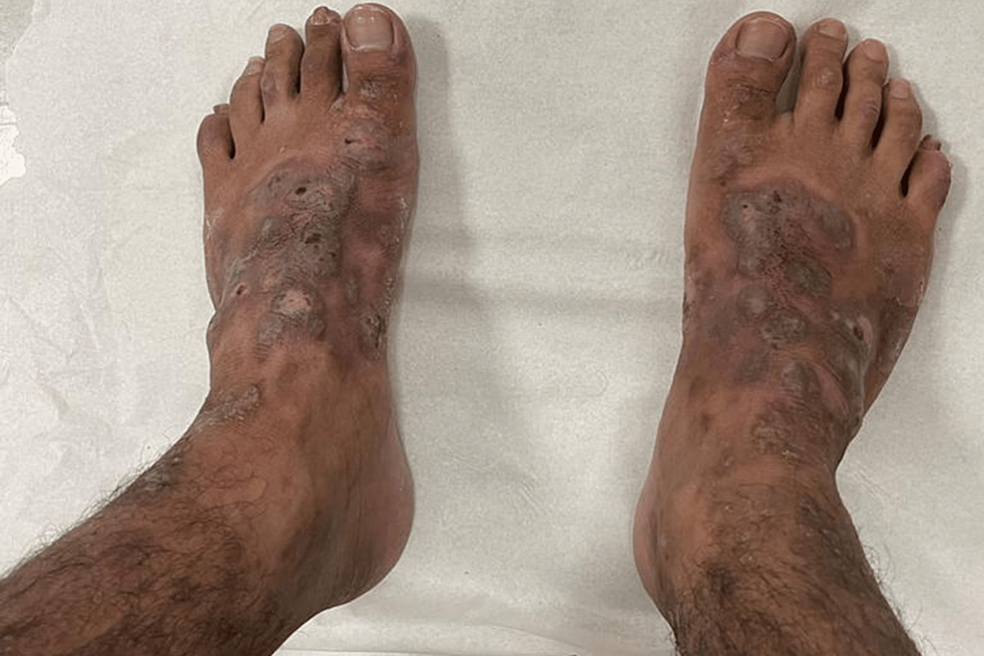
Multiple scaly, hyperkeratotic nodules and excoriations over the dorsal surface of feet

**Figure 3:**
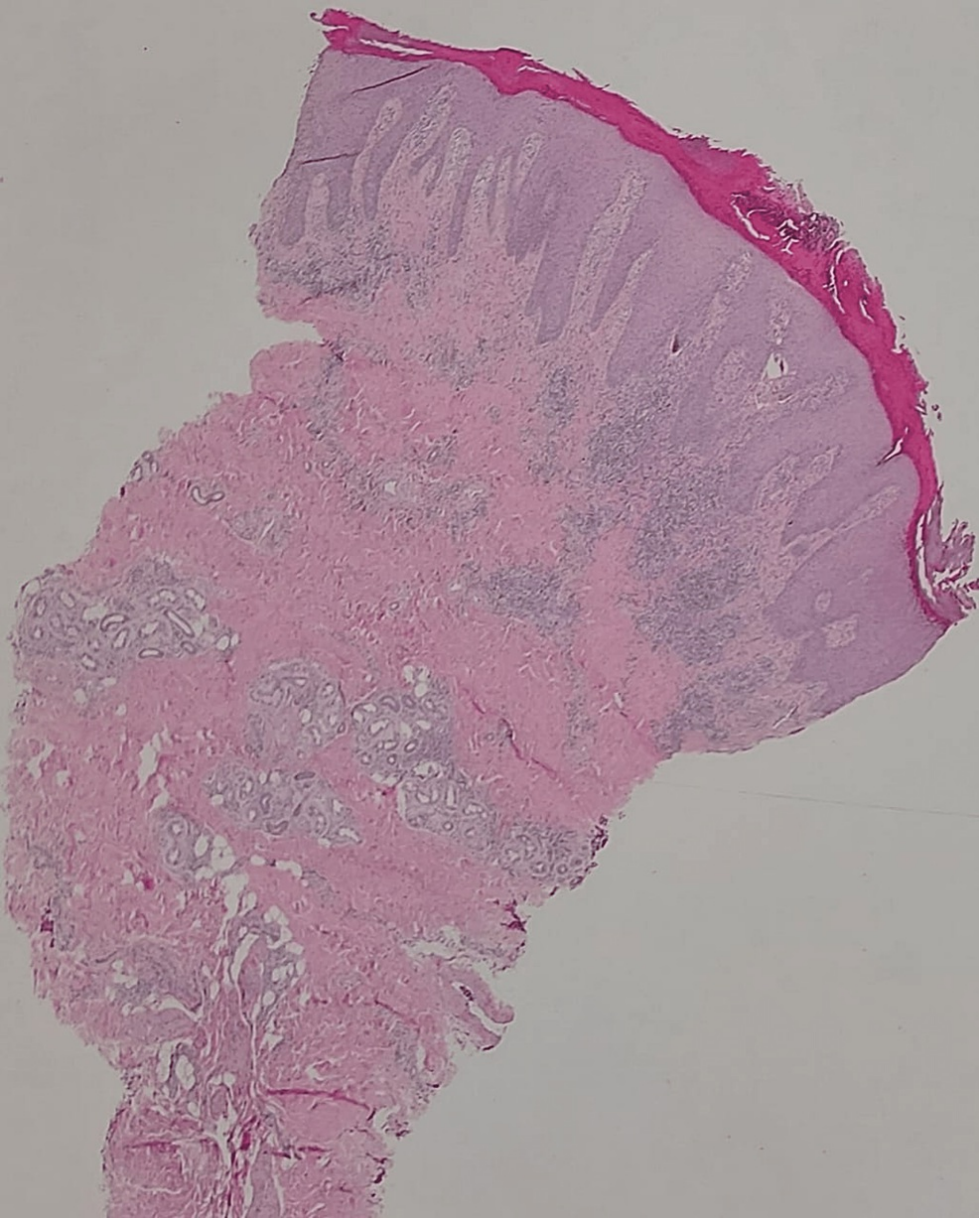
Hematoxylin and eosin stain showing a photomicrograph of low power view revealing hyperkeratosis, irregular acanthosis, and papillary dermal fibrosis (original magnification, 40x)

**Figure 4:**
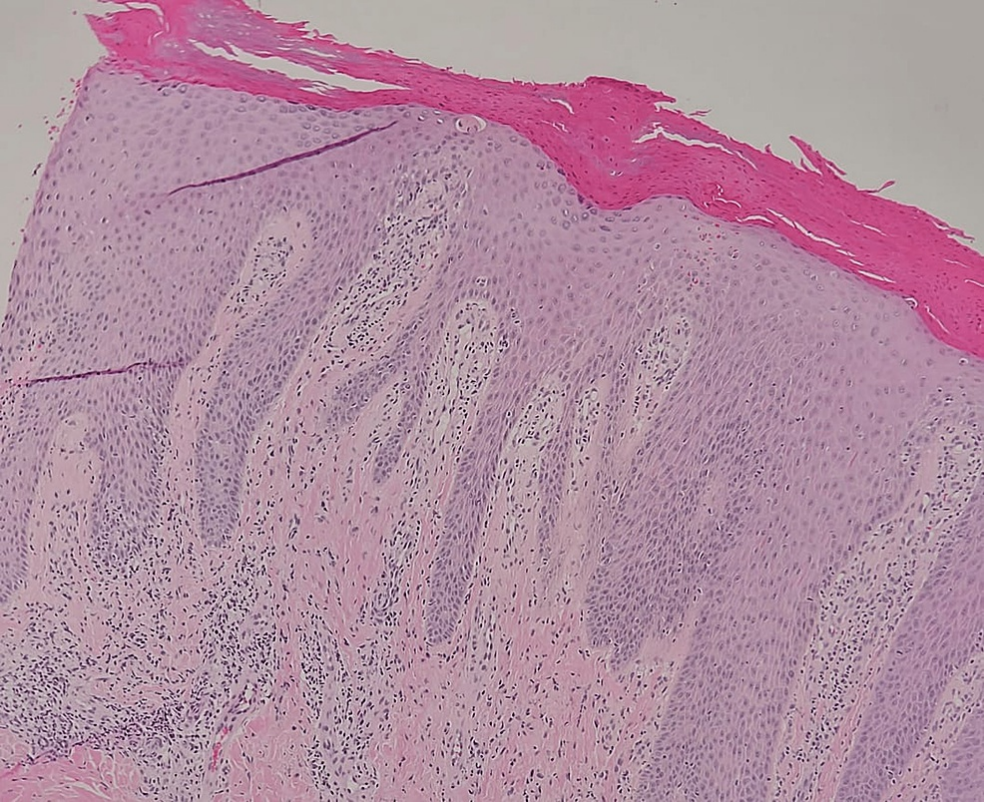
Hematoxylin and eosin stain with a higher power view showing elongation of rete ridges and superficial perivascular lymphocytes (original magnification, 200x)

A 26-year-old male presented to the outpatient clinic with itchy skin lesions over his hands and feet for five years. Physical examination showed multiple well-defined, scaly, hyperpigmented nodules surrounded by violaceous borders with erosions over the dorsum of hands and feet (as shown in Figures 1, 2) and no lesions over the trunk or other areas of the body. He was managed first by clobetasol propionate 0.05% ointment and sodium fusidate 2% ointment two times a day, and levocetirizine 5 mg tablets at night with slight improvement. Past medical history was significant for Crohn’s disease for six years on adalimumab 40 mg subcutaneously weekly, with no history of atopy or psychiatric illness. Workup for the patient was done, including complete blood count, liver function tests, renal function test, C-reactive protein (CRP), erythrocyte sedimentation rate (ESR), and viral hepatitis B and C screening, all of which were normal. The differential diagnosis was PN, hypertrophic lichen planus, and Kaposi sarcoma. A skin biopsy was taken from the left foot lesion. The result showed parakeratosis, acanthosis, and papillary dermal fibrosis (Figure 3) with elongation of rete ridges and superficial perivascular lymphocytes (Figure 4). There was no evidence of lichen planus, which supports the diagnosis of PN. After the diagnosis was made, cyclosporine 350 mg orally daily was started. The lesions and pruritus improved on cyclosporine. However, due to unexpected exacerbation of diarrhea and other gastrointestinal symptoms, the gastroenterology consultant held the medication.

## Discussion

It is well known that PN presents as itchy nodules that symmetrically affect the trunk and the extensors. However, our patient developed PN lesions over the hands and feet only (as seen in Figures 1, 2), sparing other body regions. Limited cases reported on the MIDLINE database described unique distributions of PN. There were only five reported cases of distinctive PN presentations. In 1993, Chiewchanvit et al. reported a case of a 40-year-old male with positive human immunodeficiency virus who developed acquired immunodeficiency syndrome associated with cutaneous cytomegalovirus infection presented as multiple PN-like lesions on both legs and feet [4]. In 2007, De et al. reported a case of a 60-year-old female with PN limited to thigh and buttock after six months of herpes zoster lesion at exact distribution [5]. In 2008, Lezcano et al. reported a unique presentation of a child aged one year and seven months with PN limited only over the axillary area symmetrically [6]. In 2017, Paul et al. reported a case of a 73-year-old male who presented with a well-circumscribed white tumor over the inner corner of the upper eyelid. The tumor was excised, and the biopsy showed PN as a definitive diagnosis [7]. In 2017, Akhtar et al. reported a case of a 19-year-old female with pruritus vulvae for three years that was resistant to treatment. Physical examination showed a dry patch measuring 0.5 x 1 cm on her vulva and a biopsy revealed PN [8]. Despite a few reported cases of unique presentations of PN, our case is the first reported case of PN with limited involvement over the acral areas only.

## Conclusions

PN is a dermatological disease that classically affects the trunk and extremities. Few cases reported unusual presentations. However, this is the first case that reported exclusively acral distribution that has been proven with histopathology. PN should be considered for any patient with complaints of itchy hyperkeratotic nodules, even in acral presentation. After this report, we hope that other cases on acral distribution will be reported in the future.
